# Quantifying the Spatial Dimension of Dengue Virus Epidemic Spread within a Tropical Urban Environment

**DOI:** 10.1371/journal.pntd.0000920

**Published:** 2010-12-21

**Authors:** Gonzalo M. Vazquez-Prokopec, Uriel Kitron, Brian Montgomery, Peter Horne, Scott A. Ritchie

**Affiliations:** 1 Department of Environmental Studies, Emory University, Atlanta, Georgia, United States of America; 2 Fogarty International Center, National Institutes of Health, Bethesda, Maryland, United States of America; 3 Tropical Public Health Unit Network, Queensland Health, Cairns, Queensland, Australia; 4 School of Public Health, Tropical Medicine and Rehabilitation Sciences, James Cook University, Cairns, Queensland, Australia; Duke University-National University of Singapore, Singapore

## Abstract

**Background:**

Dengue infection spread in naive populations occurs in an explosive and widespread fashion primarily due to the absence of population herd immunity, the population dynamics and dispersal of *Ae. aegypti*, and the movement of individuals within the urban space. Knowledge on the relative contribution of such factors to the spatial dimension of dengue virus spread has been limited. In the present study we analyzed the spatio-temporal pattern of a large dengue virus-2 (DENV-2) outbreak that affected the Australian city of Cairns (north Queensland) in 2003, quantified the relationship between dengue transmission and distance to the epidemic's index case (IC), evaluated the effects of indoor residual spraying (IRS) on the odds of dengue infection, and generated recommendations for city-wide dengue surveillance and control.

**Methods and Findings:**

We retrospectively analyzed data from 383 DENV-2 confirmed cases and 1,163 IRS applications performed during the 25-week epidemic period. Spatial (local k-function, angular wavelets) and space-time (Knox test) analyses quantified the intensity and directionality of clustering of dengue cases, whereas a semi-parametric Bayesian space-time regression assessed the impact of IRS and spatial autocorrelation in the odds of weekly dengue infection. About 63% of the cases clustered up to 800 m around the IC's house. Most cases were distributed in the NW-SE axis as a consequence of the spatial arrangement of blocks within the city and, possibly, the prevailing winds. Space-time analysis showed that DENV-2 infection spread rapidly, generating 18 clusters (comprising 65% of all cases), and that these clusters varied in extent as a function of their distance to the IC's residence. IRS applications had a significant protective effect in the further occurrence of dengue cases, but only when they reached coverage of 60% or more of the neighboring premises of a house.

**Conclusion:**

By applying sound statistical analysis to a very detailed dataset from one of the largest outbreaks that affected the city of Cairns in recent times, we not only described the spread of dengue virus with high detail but also quantified the spatio-temporal dimension of dengue virus transmission within this complex urban environment. In areas susceptible to non-periodic dengue epidemics, effective disease prevention and control would depend on the prompt response to introduced cases. We foresee that some of the results and recommendations derived from our study may also be applicable to other areas currently affected or potentially subject to dengue epidemics.

## Author Summary

Global trends in population growth and human redistribution and movement have reshaped the map of dengue transmission risk, exposing a significant proportion of the world's population to the threat of dengue epidemics. Knowledge on the relative contribution of vector and human movement to the widespread and explosive nature of dengue epidemic spread within an urban environment is limited. By analyzing a very detailed dataset of a dengue epidemic that affected the Australian city of Cairns we performed a comprehensive quantification of the spatio-temporal dimensions of dengue virus epidemic transmission and propagation within a complex urban environment. Space and space-time analysis and models allowed derivation of detailed information on the pattern of introduction and epidemic spread of dengue infection within the urban space. We foresee that some of the results and recommendations derived from our study may also be applicable to many other areas currently affected or potentially subject to dengue epidemics.

## Introduction

Dengue is a mosquito-borne infection that has re-emerged as a major international public health concern over the last four decades [1], [2], [3]. Caused by four closely related yet antigenically distinct single-stranded RNA viruses (genus *Flavivirus*, family *Flaviridae*), dengue viruses persist in a horizontal *Aedes aegypti*-human transmission cycle [4]. Transmission and spread of dengue infection are determined by the interplay of multiple factors including the level of herd immunity in the human population; virulence characteristics of the circulating viral strain; temperature and rainfall; survival, abundance, dispersal and blood feeding behavior of female *Ae. aegypti*; and human density, age structure, and behavior [5], [6]. As a consequence, contact rates between humans and mosquitoes are not random, but highly clustered in space and time [7].

The increasing trends in human population growth and urban redistribution that occurred over the past 40 years, coupled with the expansion of commercial trade and the rapid movement of humans (by air travel), have reshaped the global map of dengue transmission risk [8], [9]. Currently, about 70 to 100 million cases of classic dengue infection are reported every year, with an estimated 2.1 million cases of life-threatening disease in the form of Dengue Hemorrhagic Fever (DHF)/Dengue Shock Syndrome (DSS) [10]. Furthermore, the number of dengue fever epidemics has increased dramatically, and an expansion of the dengue endemic and hyperendemic areas is indisputable [2], [8], [9]. Under current socio-epidemiological scenarios, continued geographic expansion of epidemic dengue is expected to continue (as observed in north and central Argentina, where dengue transmission was registered for the first time in 2009 [11]), and severe dengue and DHF outbreaks are expected to follow once mixing of multiple serotypes occurs. Dengue epidemics in such areas are commonly originated by viremic travelers from endemic regions [9], and can cover large areas leading to a large number of cases as a consequence of the limited (or null) population exposure to dengue viruses, the prevailing high vector abundances and the challenges faced by local vector control programs on dealing with massive outbreaks.

Consistent with global trends, outbreaks of dengue have become more frequent and severe in Australia, occurring exclusively in north Queensland (NQ) [12], [13], [14], [15], [16], [17]. Originated by viral introduction via infected travelers from endemic regions, dengue outbreaks in this region are characterized by a rapid spread both in time and space, as a result of the prevailing high *Ae. aegypti* populations [18] and the movement of residents and tourists within and between urban centers [15]. The recent occurrence of two major and widespread epidemics in 2003 and 2008–2009 has challenged local health authorities with the question of whether NQ will join the growing list of dengue endemic regions.

In Queensland, the Tropical Population Health Unit (TPHU) dependent of Queensland Health is the institution responsible for regular vector and viral surveillance, vector control, outbreak response, public education, and operational research [12], [19]. In late February 2003, TPHU was notified of three locally acquired dengue fever cases in a small industrial area in the city of Cairns [15]. Mosquito-control measures were implemented immediately and local doctors were alerted. These cases were quickly confirmed as dengue virus serotype 2 (DENV-2) [15]. Vector-control measures began in the affected Cairns neighborhoods following the Dengue Fever Management Plan for north Queensland (Supporting Information S1), and extended until the outbreak was declared over, five-and-a-half months and 459 cases (383 of which occurred in the city of Cairns) after the primary introduction [15]. In following up these cases, it was learned that a PNG national introduced the virus into Cairns in late January soon after arriving from PNG into a neighborhood heavily infested by *Ae. aegypti* [18]. In support of such case as the epidemic's putative index case are the following: a) dengue is not endemic in Cairns, indeed, there was no confirmed dengue activity in urban Cairns before the IC arrival into Cairns from PNG; b) once transmission was confirmed in the area, the sera of the putative case (originally misdiagnosed as malaria) was retrospectively confirmed by PCR as DENV-2 positive, 49 days after its arrival from PNG [15]; c) the DENV-2 from 2003 had 99.8% homology with an import from PNG into the nearby city of Townsville in April 2003 [15], indicating PNG as the most likely origin of the DENV-2 introduction; d) most of the early cases occurred around the putative index case's residence.

Dengue infection spread in naive populations occurs in an explosive and widespread fashion [5], [20], [21], [22], [23] as a consequence of the combination of the lack of herd immunity, the population dynamics and dispersal of *Ae. aegypti* and the movement of individuals within the urban space [15], [24]. Knowledge on the relative contribution of such factors to the pattern of virus propagation during epidemic events has been limited. As a consequence, the spatial dimension of dengue virus spread within complex urban environments is unknown. Although a few studies had quantified the spatial pattern of dengue epidemic transmission in urban environments [20], [22], [23], [25], [26], difficulties in assessing where the virus has been first introduced had limited their description of the pattern of dengue infection spread. In the present study we analyzed the pattern of spread of the DENV-2 outbreak that affected the city of Cairns (NQ) in early 2003, quantified the relationship between dengue spread and the location of the epidemic's index case (IC), derived a dispersal kernel for virus spread, assessed the effects of vector control in the containment of the infection, and generated recommendations for city-wide improvement of dengue surveillance and control.

## Materials and Methods

### Study area

The city of Cairns (total 2006 population: 140,347) is located in the wet tropics of northeastern Queensland, Australia (16.9° S; 145.8° E). Cairns has a tropical monsoonal climate, with respective mean daily low and high temperatures of 18°C and 26°C in winter and 24°C and 31°C in summer, and most of the rainfall (ca. 82% of the annual 1,992 mm) falling during January–April (Australian Bureau of Meteorology). Residential areas consist of a mixed housing type, with relative small 1–3 story apartment blocks interspersed with single family houses. Housing ranges from modern brick, concrete block and stucco structures to wooden houses that are 50–100 years old. These old wooden ‘Queenslander’ houses are elevated on wooden or concrete poles and typically feature unscreened windows to maximize air flow. Older suburbs of Cairns (i.e., Parramatta Park, Manuda, Cairns North, Edge Hill) contain mostly ‘Queenslander’ houses, and often are surrounded by dense tropical vegetation. Cairns also is surrounded by a series of small isolated ‘beach communities’ that abut the Coral Sea. The present study focused on dengue transmission dynamics in urban Cairns; beach communities and satellite towns were excluded because of their low (or absence of) dengue transmission [17].

### Data sources and management

De-identified data with age, sex, date of onset of infection, and geographic position of the most likely place of dengue infection for each of the 383 laboratory-confirmed human dengue cases that occurred within urban Cairns in 2003 were provided by TPHU. Additional information given by TPHU included: location and timing of each indoor residual spray (IRS) performed by the Dengue Action Response Team (DART), GIS layers with information on the road network and each one of the 32,716 premises censused in urban Cairns in the year 2003, and a 2004 orthorectified high-resolution satellite image (Ikonos, GeoEye, Dulles, VA) of the city of Cairns. All geographic layers were processed in a Geographic Information System (ArcGIS 9.3, ESRI, Redlands, CA) in order to hide their absolute location while preserving the relative distance between them (to protect each patient's identity).

Dengue is a notifiable diseae in Australia [19], [30]. Upon suspicion of a dengue case, medical practitioners from private clinics and the Cairns hospital are required to contact TPHU to provide details about each case (name, address, phone numbers and specific symptoms; see Figure S1 in Supporting Information S1 for a sample notification form). Also, all pathology laboratories in Queensland are required to promptly notify public health authorities of any laboratory result indicative of a recent dengue infection (i.e., positive dengue IGM titers or virus detection by PCR). Within 24 hours of receipt of a notification of a suspicious or a laboratory confirmed case highly trained TPHU public health nurses perform contact tracing telephonic interviews to determine a patient's travel history and to identify the origin of infection (i.e., imported or locally acquired dengue), the date of onset of infection (i.e., date of onset of symptoms minus the intrinsic incubation period of 4–7 days [5]), the locations a patient visited while viremic and, ultimately, the most likely place where infection has occurred [30]. Refer to Figure S2 in Supporting Information S1 for a sample dengue case report form used by TPHU nurses to obtain information on each case and to infer the most likely place of infection.

The most likely place of infection of each case was determined by epi-linking the places visited during the 4–7 days before the onset of illness. The locations were based upon a) home address, b) work address, or c) places and people who they have visited during the exposure period (Figure S2 in Supporting Information S1). Patients were asked for addresses where they had been bitten by mosquitoes during the exposure period. Any addresses with recent (∼2–4 weeks) dengue activity were considered a likely place of infection. The selection of the most likely place of infection for cases epi-linked to more than one premise was based on the amount of daytime spent by a person on each location (i.e., the premise in which a patient spent most of his/her daytime was selected as the exposure site). The information of the most likely place of infection for cases without any epidemiological link to transmission areas (based on the phone interviews) was updated if new information, such as the confirmation of previously unknown dengue cases within 2 weeks of the onset of symptoms of the initial case, was obtained. Interviews were also performed with the persons each patient identified as potential primary or secondary contacts (e.g., work mates, relatives, friends) to pre-empt the detection of further secondary infections. Based on estimates of a recent (2008–2009) epidemic, TPHU nurses generally interview approximately 3 potential cases per every confirmed case (D. Brookes, Queensland Health, personal communication). Given that TPHU only kept digital records of the most likely places of dengue infection for each case (after the change from paper to digital records, TPHU discarded all phone interview paper records) in this study we were unable to analyze the accuracy of nurses in identifying them. While we acknowledge that some of the dengue acquisition addresses may have been incorrectly identified during the interviewing process (due to patients' recall bias), we believe that most are accurate and that the general spatial nature of the outbreak is not radically perturbed by the misidentification of a small number of acquisition addresses.

Upon confirmation of an address where a patient spent time while viremic, TPHU initiated vector control activities as described in Supporting Information S1. The date and address for every IRS/source-reduction intervention were recorded in a geodatabase linked to the Cairns GIS. In the present study we only analyzed data on indoor residual spraying (IRS) because, when properly applied, it represents an effective method to supress *Ae. aegypti* indoor populations [31].

We estimated the age-adjusted incidence of infection for the 2003 epidemic by applying the direct method [32]. This method adjusts the amount that each age group contributes to the overall rate of transmission, so that the overall rates are based on the same age structure [32]. Such adjustment was calculated by first multiplying the age-specific disease rates by age-specific weights (i.e., the proportion of the standard 2000 Queensland population within each age group) and then summing the weighted rates across all age groups to give the overall age-adjusted dengue incidence rate for the 2003 epidemic. In this way, we eliminated possible interferences in the estimates due to heterogeneous demographic structure of the studied population.

### Data analysis

Classic, spatial, and Bayesian statistical analyses were performed to describe and quantify the spread of dengue in the 2003 Cairns epidemic. A maximum likelihood logistic regression [32] was employed to assess the impact of IRS on the odds of secondary dengue infections at the house level. Particular attention was paid to the timing of spray versus the occurrence of cases in a house, since variation in the incubation period of dengue (∼7 days when accounting for intrinsic incubation period) can affect the predicted relationship between spraying and dengue cases (by having cases infected before the spraying but showing symptoms after the spraying). Cross-correlation time series analysis [33] between the weekly number of cases and the weekly number of IRS applications, weekly precipitation and average temperature were performed to assess the relationships between the temporal progression of the epidemic and selected environmental factors. Meteorological data for Cairns was obtained from the Australian Bureau of Meteorology (source station: Cairns International Airport, located <4 km from the introduced case's residence).

Spatial analyses were performed to quantify the intensity (Local K-function) and directionality (angular wavelet analysis) of the spatial clustering of dengue cases around the introduced case (IC), and space-time analysis (Knox test) were applied to identify independent space-time clusters of dengue transmission during the 25 weeks of the outbreak. The Local K-Function (*L_i_(d)*) developed by Getis and Frankling [34] was applied to determine the overall distance up to which cases clustered around the IC. In our focal analysis of *L_i_(d)*, *i* is represented by the location of the IC. Statistical inference of *L_i_(d)* is performed by comparing the observed *L_i_(d)* with the expected *L_i_(d)* generated by 999 Monte Carlo realizations under the hypothesis of Complete Spatial Randomness [34]. A focal spatial correlogram of *L_i_* over *d* was used to assess the distance up to which clustering of dengue cases around the IC was maximized (*d_max_*). Briefly, *d_max_* represents the distance at which the value of *L_i_* is maximum. *d_max_* is interpreted as the distance beyond which the inclusion of further cases does not increase the probability that the distribution of events differ from random [34].

To determine if the distribution of cases around the IC occurred predominantly in a given cardinal direction (i.e., anisotropic distribution) we divided the space around the IC's house into one degree sectors, and counted the number of cases that fell within each sector. These counts were then analyzed by angular wavelet analysis [35]. A wavelet function *g*(*x*) is a scalable windowing function. In our study we used the French Top Hat [35] as a wavelet function. The main metric derived from fitting the wavelet function to the data is the wavelet positional variance. Peaks in this variance indicate directions where most of the cases fell relative to the IC. In order to separate true patterns from random fluctuations, the significance of the wavelet analysis was determined by comparing the observed variance with the one obtained from 999 Monte Carlo simulations [35]. The analysis was performed for both the location of cases and the location of Cairns houses to determine if any anisotropic pattern in the distribution of cases could have been an artifact of the spatial arrangement of houses within the city. Visualization of spatial anisotropy in the occurrence of cases was performed by estimating spatial standard-deviation ellipses in ArcGIS 9.3 (ESRI).

The Knox method [36] was applied to quantify the space-time interaction of individual confirmed dengue cases reported during the outbreak. This method tests for possible interaction between the distance and time separating cases, based on the number of case pairs found in a particular time-space window (e.g., case pairs separated by less than *M* meters and *T* days) [36]. When interaction is present, distances between pairs of cases will be small, and the test statistic will be large. In our study we chose the values of *M* and *T* as 100 m and 20 days, respectively, to account for mosquito dispersal distance (i.e., *Ae. aegypti* seldom disperses beyond 100 m [37], [38]) and virus incubation periods (i.e., maximum sum of intrinsic and extrinsic incubation periods). The expected values of the test under the null hypothesis of random case occurrence (in space and time) were estimated by performing 999 Monte Carlo simulations.

A semiparametric Bayesian space-time structured additive regression model (STAR) [27], [39] was applied to assess the effects of IRS, rain and spatial autocorrelation in the odds of weekly dengue infection over the first 15 weeks of the epidemic. Since our analysis focused on weekly data we selected a discrete-time duration STAR model [27]. Such model allows a unified treatment of time scales, linear and non-linear effects of covariates and spatially correlated random effects within a Bayesian framework [27]. Briefly (refer to [27] for a detailed description), a space-time geoaditive STAR model has the following predictor structure:
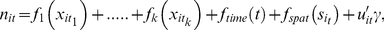
(1)where *u*′_it_γ are the usual linear predictors (fixed effects) for covariate vector *u*, *f*_1..k_ are possibly non-linear functions of the covariates, *f*_time_ is a possibly non-linear time trend and *f*_spat_ is a spatially correlated (random) effect of the location *s*_it_ an observation pertains to [27].

In a Bayesian approach, all non-linear functions *f*_j_ and parameters γ_i_ are considered as random variables and (1) is therefore conditional upon these random variables, having to be supplemented with appropriate prior distributions. Fixed effects were modeled by independent diffuse priors (*p(γ_j_)∼const*, *j* = 1, …, r), whereas the priors for unknown functions (*f*_j_) were dependent on the type of covariate and on the prior knowledge about smoothness. The fixed effects (*f*_1..k_) and the time function (*f*_time_) were modeled via Bayesian penalized splines (P-splines), whereas Markov random field priors were chosen as priors for the spatial effects (*f*_spat_) [27]. Posterior means together with confidence intervals and other parameters are obtained by drawing samples from the posterior by Markov chain Monte Carlo (MCMC) techniques [27]. Variance parameters (*τ_j_^2^*) can be estimated by assigning additional hyperpriors to them. The most common assumption is that *τ_j_^2^* are independently inverse gamma distributed, i.e., *τ_j_^2^∼IG(a_j_,b_j_)*, with hyperparameters *a_j_* and *b_j_* a priori set as *a_j_* = *b_j_* = 0.001. For updating the parameters in a MCMC sampler, a Metropolis-Hastings algorithm based on iteratively weighted least squares was applied [27]. We performed a sensitivity analysis of the best fitted model by varying the values of *a_j_* and *b_j_* between 1 and 0.0001 as described in [39].

Our STAR model evaluated the effect of rain, the cumulative proportion of sprays performed around a premise (cum_spr) and the spatial arrangement of premises (spat) on the odds of weekly dengue virus infection (binomial variable, 0 = no infection, 1 = infection) at the house level from weeks 0 to 15 post introduction. Analysis focused in the neighborhoods surrounding the IC's house (Parramatta Park, Manuda and Cairns North), where most of the cases and IRS applications occurred. Thyssen polygons from the centroids of all the premises within the analyzed area were generated to allow contiguity weighting schemes for the spatial random effects. Our analysis matrices had a dimension of 1,490 premises by 15 weeks (or 22,350 repeated measurements), and the structure of the evaluated models was as follows:
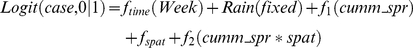
(2)and
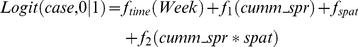
(3)We compared both models in their Deviance Information Criterion (DIC, the AIC equivalent for Bayesian models [27]) after 100,000 MCMC randomizations, and only reported the results of the model with the lowest DIC.

Spatial and spatio-temporal analysis were performed with ClusterSeer 2.0 (Terraseer, Ann Arbor, MI), R 2.10.1 (http://www.r-project.org/) and ArcGIS 9.3 (ESRI) software, whereas Bayesian STARS were performed with BayesX software (http://www.stat.uni-muenchen.de/~bayesx) and model outputs visualized with R package BayesX (http://cran.r-project.org/web/packages/BayesX/).

### Scientific and ethical review and approval

The protocols for storing, analyzing and reporting results on the 2003 Cairns dengue epidemic data were approved by Queensland Health's Human Research Ethics Committee (protocol HREC/09/QCH/52-590).

## Results

### Epidemic progression and control

**Figure 1:**
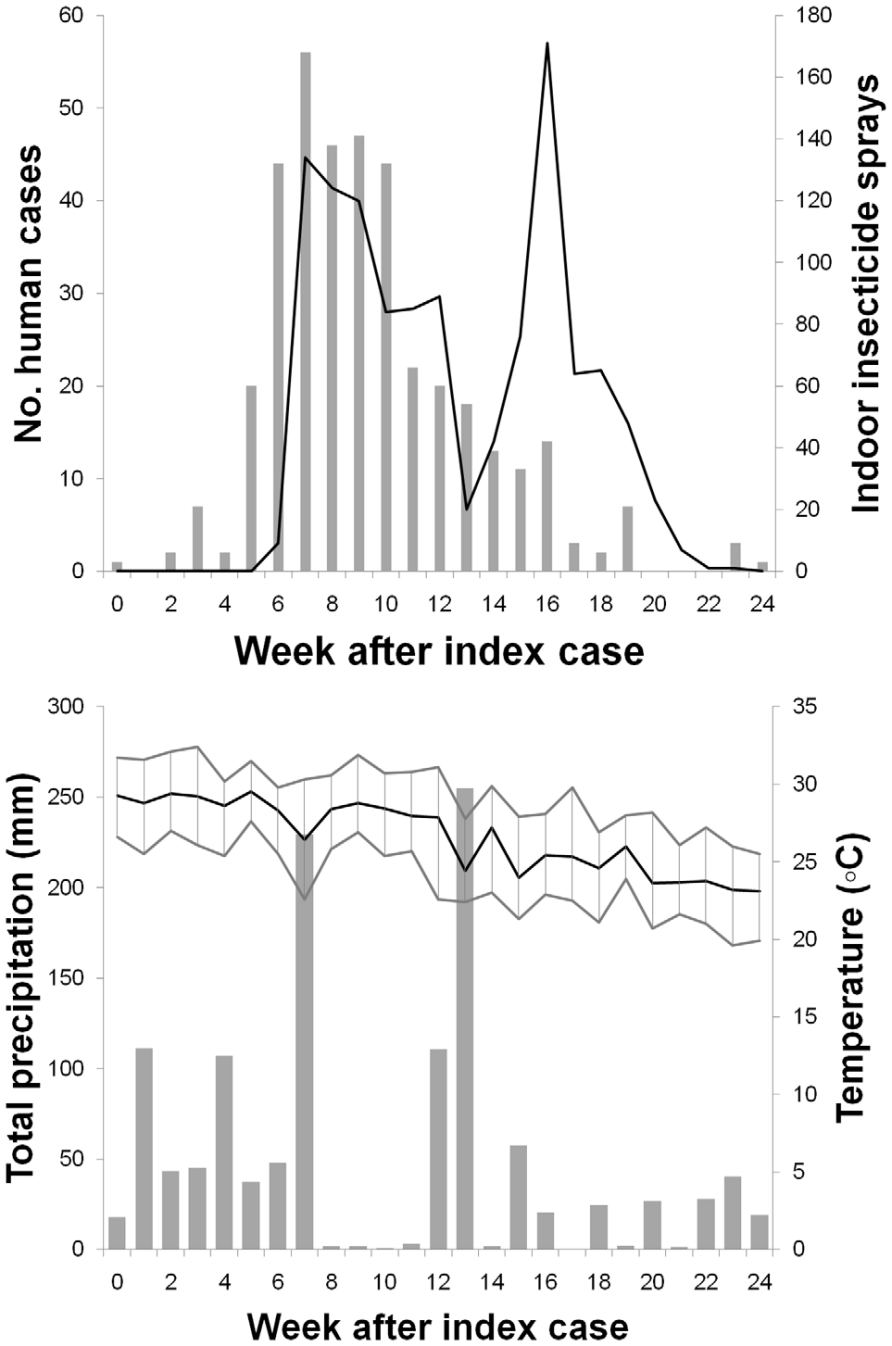
The 2003 Cairns epidemic in numbers. (A) Weekly number of confirmed dengue 2 cases (bars) and of indoor residual insecticide sprays (line) performed in the city of Cairns during January–August 2003, and (B) weekly variation in temperature (mean, minimum and maximum) and total precipitation over the same period. Time is measured in weeks since the onset of symptoms of the introduced case (IC).

Local transmission of dengue in Parramatta Park began 18 days after the onset of symptoms in the imported case (21 January). However, the TPHU was not notified of dengue activity in the area until 5 March (∼43 days post-introduction) and initiated mosquito control measures the next day. It took ∼49 days to retrospectively confirm that the traveler from PNG (initially misdiagnosed with malaria) was indeed the epidemic's IC. A total of 383 laboratory-confirmed symptomatic cases were registered within urban Cairns over the 25-week epidemic period that followed the initial introduction. All infections were confirmed as mild (only 5% of all cases were hospitalized) DENV-2 derived from the initial introduction; no dengue-related deaths or DHF manifestations were reported. The index case (week 0) and subsequent first wave of local transmission (weeks 2–3) are clearly distinguished by the epidemic curve (Figure 1A). The weekly number of confirmed dengue cases then showed an exponential growth from one to 56 cases from weeks 4 to 7 post introduction (PI), followed by a stable period ranging from 44 to 47 cases during weeks 8–10 PI, and a final exponential decay thereafter (Figure 1A). The weekly pattern of IRS applications followed the pattern of human cases, totaling 1,163 applications over a 19 week period (Figure 1A). The overall age-adjusted incidence rate of the 2003 Cairns epidemic was 1,148 cases per 100,000 (Table 1). The highest incidence rates were observed in males 30–39 year old. Overall, young adults (30–39 years old) presented the highest incidence rates (average [SD], 258.5 [27.9] and 193.6 [16.9] cases per 100,000 for men and woman, respectively), but not in the younger or older ages (Table 1).

**Table 1:** Adjusted dengue incidence rates per age class for the city of Cairns during January–August 2003.

	No. cases			Age adjusted incidence (cases per 100,000)		
Age group	Males	Females	Total	Males	Females	Total
0–9	4	7	11	30.4	52.1	41.4
10–19	23	19	42	189.1	153.9	171.6
20–29	57	50	107	255.4	213.2	233.8
30–39	53	28	71	287.8	183.2	241.8
40–49	37	27	64	232.3	184.5	209.1
50–59	28	26	54	173.6	174.7	173.9
60–69	10	4	14	63.9	27.7	46.3
≤70	4	6	10	25.3	34.8	30.6
Total	216	167	383	1,257.7	1,024.2	1,148.4

The weekly number of cases was strongly and positively correlated (Cross-correlation coefficient, r>0.6) with the number of IRS applications up to a time lag of 2 weeks (Figure S3 in Supporting Information S1). Such positive correlation was consequence of the strong and active response of TPHU to the DENV-2 epidemic once DENV-2 transmission was confirmed (Figure 1A). The weekly variation in temperature (mean, minimum and maximum) and total precipitation during the 25-week period of the outbreak (Figure 1B) did not show any strong correlation (r<0.5) with the weekly number of cases (Figure S3 in Supporting Information S1). However, the lack of rain during weeks 8–11 (Figure 1B) coupled with the high intensity of IRS applications during that period could have acted synergistically against local *Ae. aegypti* populations and help explain the sharp reduction and further interruption of dengue transmission after week 11 PI (Figure 1A).

### Spatio-temporal pattern of spread

**Figure 2:**
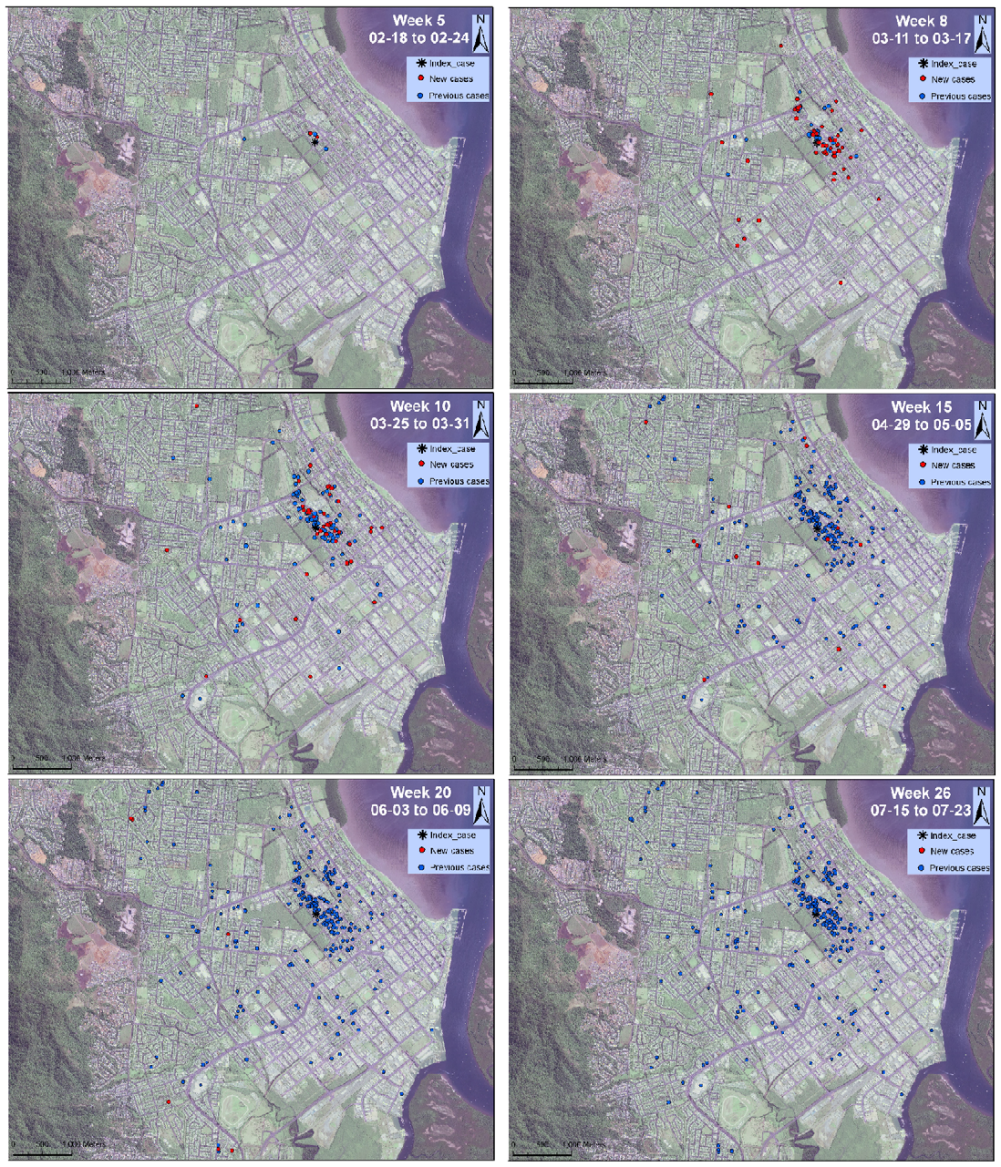
Spatial progression of the dengue epidemic that affected the city of Cairns, Australia, during January–August 2003. Time is represented as weeks since the onset of symptoms of the introduced case (IC). Red circles represent cases confirmed on the week shown, whereas blue circles represent the cumulative cases until that occurred up to that week. Video S1 is an animated version of this figure.

Figure 2 (and Video S1) depicts the spatio-temporal pattern of dengue spread in Cairns during the 25-week transmission period. Up to week 5 PI, most transmission was contained around the IC (all but one confirmed case occurred within 100 m of the IC). Within the first 3 weeks PI, two additional cases occurred in the same house of the IC. Between weeks 6 and 10 PI, transmission continued propagating around the IC, but also expanded to other neighborhoods located further than 100 m of the IC. An increase in the distance of new cases to the IC together with a reduction in the number of confirmed cases were evident by week 15 PI. Between weeks 15 and 25 PI only a few isolated cases occurred, most of them located in the periphery of urban Cairns. The last case was contracted on week 25, and the epidemic was formally declared over three months later.

**Figure 3:**
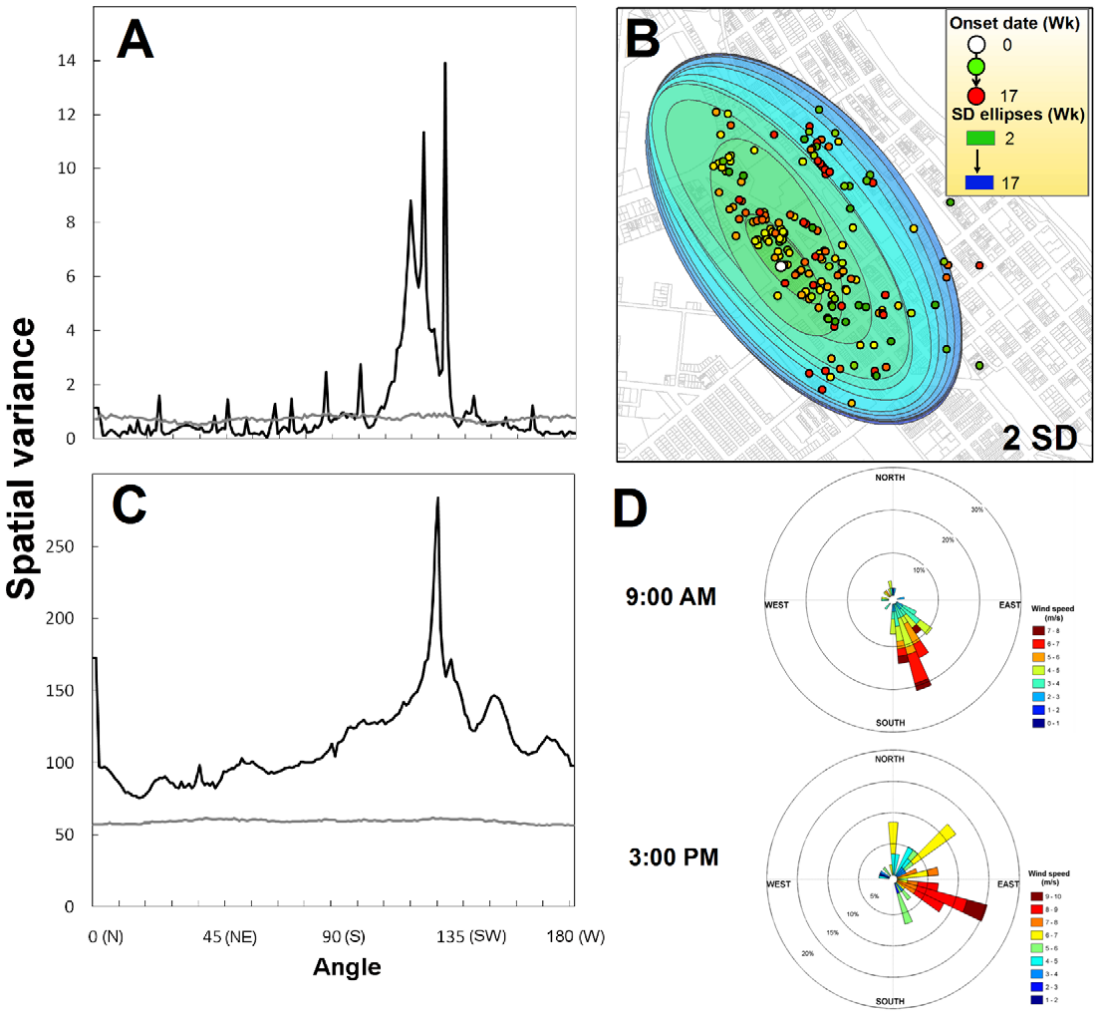
Directionality analysis for the location of dengue cases within *d_max_* (the distance up to which clustering of cases around the introduced case (IC) occurred). (A) Wavelet directionality analysis (WDA) for dengue cases. (B) Standard deviation (SD) ellipses showing the spatial anisotropy of dengue cases. (C) WDA for houses. (D) Wind rose plots showing the directionality (measured in 20 degree bins from north cardinal point) of daily prevailing wind direction and speed at 9:00 AM and 3:00 PM during February–March 2003 (the first 2 months of the epidemic, when most dispersal around the index case occurred).

Focal clustering of DENV-2 cases was maximized at a distance of 800 m from the IC (*L_i_(d_max_)* = 1,742.3; *P*<0.05; Figure S4A in Supporting Information S1). A total of 240 cases (63%) were located within *d_max_*, mainly in the neighborhoods Parramatta Park (PP) and Cairns North (CN) (Figure S4B in Supporting Information S1). The spatial wavelet directionality analysis performed within *d_max_* showed a significant peak (Spatial Variance >1.96; *P*<0.05) in the orientation of cases around the IC towards the NW-SE direction (Figure 3A,B). A significant peak in the distribution of houses around the IC towards the same NW-SE direction (Figure 3C) points to the orientation of the built environment (blocks were aligned NW to SE and elongated at a ratio of ca. 3× longer than wide) as the most likely cause of the anisotropic distribution of cases around the IC. The dominant wind direction during the first 2 months from the initial epidemic spread from the index case (Feb.–March) was from the SE quadrant (ie., 60–180°; Figure 3D), with 80% and 44% of winds at 9 AM and 3 PM from such quadrant, respectively. Such prevailing early morning and late afternoon winds could have affected dispersal of infected *Ae. aegypti* females, also contributing to the SE/NW oriented anisotropic distribution of dengue infections around the IC (Figure 3B).

**Figure 4:**
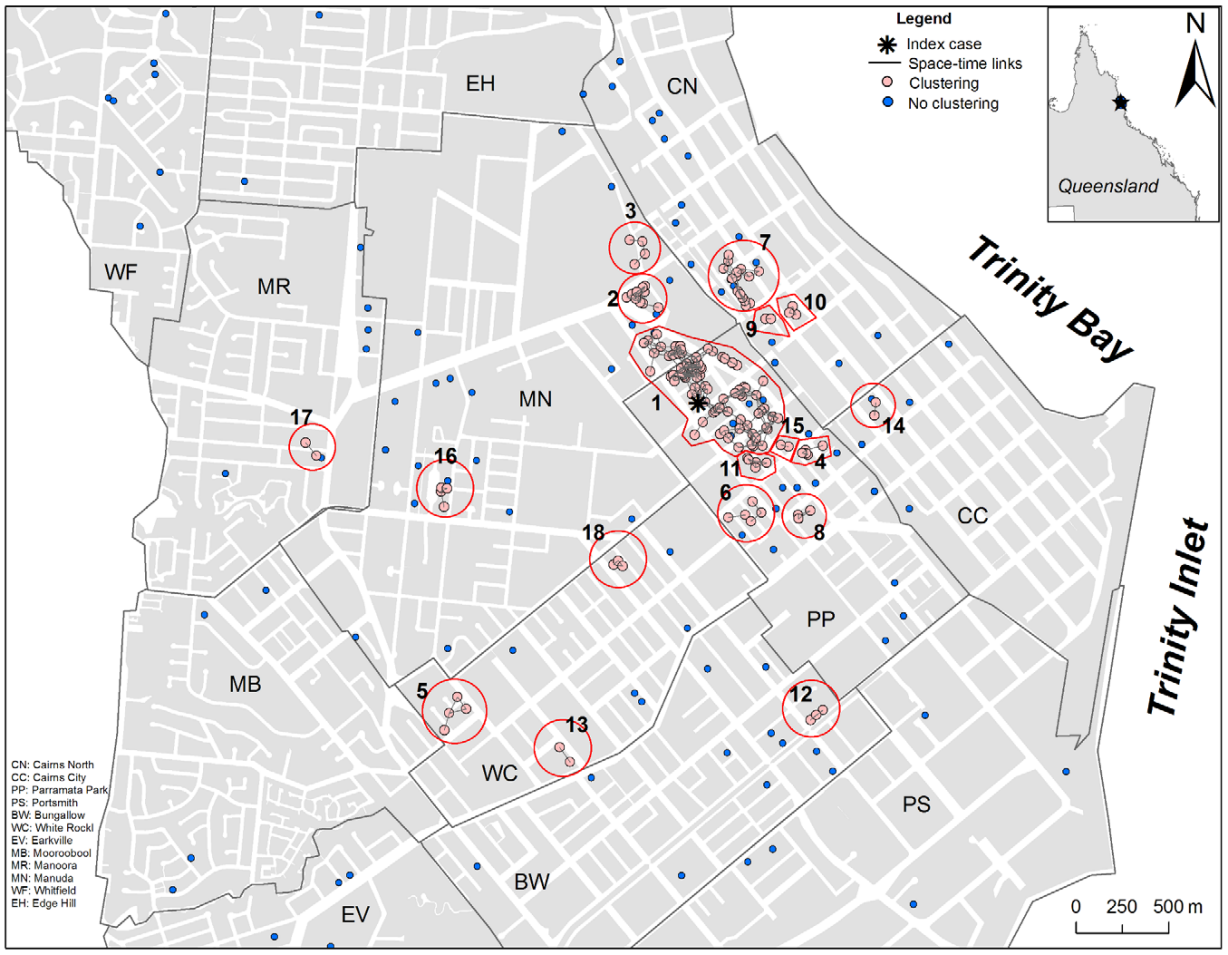
Significant space-time clustering (assessed by the Knox test) of dengue cases in the city of Cairns, Australia, during January–August 2003. Red circles and numbers identify each individual space-time cluster. Detailed information about each cluster can be found in Table 2.

The space-time Knox test statistic identified a total of 18 independent significant space-time clusters involving 250 (65.3%) cases (Figure 4). The remaining 133 (34.7%) cases did not show any spatio-temporal association among them or with members of the space-time clusters. Table 2 summarizes the characteristics of each identified space-time cluster, together with the average distance of each case to the original IC and the putative index case (PIdC) of each cluster, and Figure S5 in Supporting Information S1 shows the weekly distribution of the proportion of cases within each space-time cluster. The earliest and largest cluster (cluster 1) occurred around the IC, and included 129 cases (including the IC) extending over 440 m during a 13-week period (Figure 4, Table 2). The second cluster was initiated 37 days after the onset of symptoms of the IC, and was located at an average distance of 679 m from the IC (Figure 4, Table 2). The average distance between every cluster's case and the case that first showed symptoms (i.e., the cluster's putative index case, PIdC) was 75 m (SD: 51.2 m) whereas the average distance between cases within a cluster was 73.2 m (SD, 59.9 m) (Table 2). The spraying response (measured in days since the onset of symptoms of a cluster's first reported case) in each cluster averaged 15.3 days (range, 0–40 days) whereas the spraying coverage averaged 36.8% (range, 0–100%) for all the premises with cases and 23% (range, 0–47.1%) for all the premises found within 100 m of a premise with a confirmed case (Table 2).

**Table 2:** Description of each space-time cluster identified for the dengue epidemic that affected the city of Cairns during January–August 2003.

Cluster code^1^	Onset first case (days since IC)^2^	No. cases	No. houses	Duration (days)	Mean distance to IC (range)	Mean distance to PIdC[Table-fn nt103] 3 [range]	Mean inter-case distance [SD]^4^	% case houses sprayed^4^	Delay spraying^5^	% houses sprayed 100 m^6^
1	0	129	81	93	220 [0–440]	220 [0–440]	285 (150)	58.0	40	37.4
2	37	30	11	48	679 [566–700]	44 [0–159]	58 (34)	0	*	2.0
3	40	6	4	20	880 [832–963]	94 [69–130]	86 (42)	0	*	2.3
4	44	7	5	26	661 [622–711]	84 [0–119]	58 (42)	60	27	28.8
5	48	4	4	17	2,133 [2,056–2,242]	151 [94–193]	121 (40)	75	15	30
6	50	7	5	20	642 [606–699]	94 [46–123]	89 (42)	40	8	14.7
7	51	18	15	40	701 [590–823]	124 [0–262]	119 (62)	33.3	19	27.0
8	54	6	3	14	824 [809–837]	35 [0–68]	44 (33)	33.3	11	40.6
9	55	7	2	40	599 [586–604]	23 [0–27]	13 (13)	100	10	35.5
10	62	2	2	3	956 [953–957]	71	71 (0)	0	*	47.1
11	69	6	5	19	445 [398–490]	44 [0–68]	54 (26)	80	0	35.5
12	69	5	3	24	1,807 [1,795–1,821]	21 [0–45]	34 (23)	0	*	0
13	70	3	2	5	2,047 [2,011–2,065]	96 [96–96]	64 (45)	0	*	0
14	73	4	3	12	710 [695–733]	41 [37]–[48]	34 (13)	33.3	24	14.0
15	80	2	2	2	520 [500–541]	42	42 (0)	50	5	41.1
16	101	6	4	22	1,466 [1,433–1,483]	45 [0–84]	59 (40)	0	*	0
17	102	3	2	3	2,099 [2,082–2,133]	92 [92–92]	61 (43)	0	*	13.0
18	111	4	3	4	972 [956–987]	36 [34]–[37]	35 (16)	100	9	44.6

1 Refer to Figure 4 for a geographic identification of each cluster.

2 Represents the number of days between the onset of symptoms of the PIdC and the onset of symptoms of the IC.

3 PIdC = putative index case of a cluster.

3 The mean distance between all cases belonging to a cluster.

4 Percentage of locations with cases that were sprayed with insecticides during the epidemic period.

5 Number of days between the onset of symptoms of the PIdC and the date a house was sprayed within the cluster.

*represents lack of sprayings.

6 Percentage of houses that were sprayed within 100 meters of all the cases belonging to a cluster.

Refer to Figure 4 for a geographic representation of each cluster.

**Figure 5:**
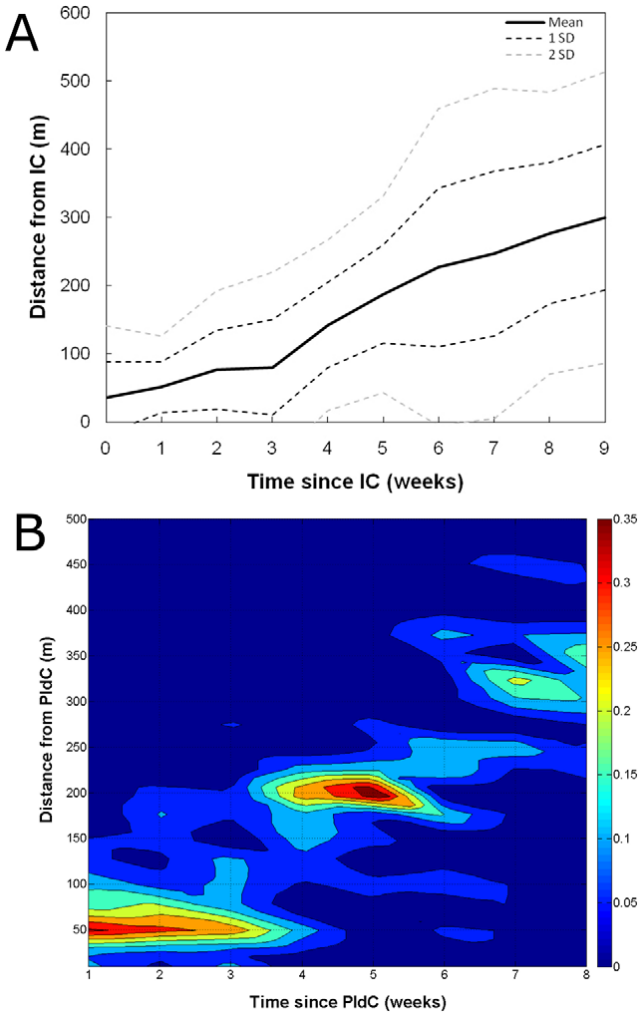
Using human infection as a marker of dengue virus epidemic spread. (A) Association between the proportion of dengue cases that occurred on a given week (colors) and the spatial and temporal distances to a putative index case (PIdC) within a space-time cluster. PIdC can be interpreted as the most likely origin of a space-time cluster. (B) Mean distance from a confirmed dengue case to a PIdC according to the time (in weeks since the onset of PIdC symptoms) such case started presenting dengue symptoms. SD represents standard deviation of the mean value.

For each space-time cluster we identified its first confirmed case and estimated the spatial and time distances from each secondary case in the cluster to this PIdC. PIdC can be interpreted one of the most likely origins of a space-time cluster. We then considered each cluster as a replicate to assess the relationship between distance and time since the onset of symptoms of a PIdC. The distance up to which most of the secondary cases were found increased with the time since the onset of symptoms of a PIdC from 50 m to 200 m and 325 m for weeks 1–3, 4–6 and 7–8, respectively (Figure 5A). Most of the cases whose onset of symptoms was within 3 weeks of the onset of symptoms of a PIdC were found within 50 m of it (Figure 5A). The mean distance from a secondary case to a PIdC increased linearly with the time after onset of symptoms of the PIdC, at a rate of 14.4 m (SD, 16.2 m) per week during weeks 1–3 and 32.9 m (SD, 13.2 m) per week from weeks 4–8 post onset of symptoms of the PIdC (Figure 5B). About 95% (2 SD) of all the cases found within the first week of onset of symptoms of the PIdC occurred 125 m around it (Figure 5B). This distance increased to 200 m by week 3. Figure 5B can be interpreted as a kernel of dengue diffusion from a PIdC after filtering out the effect of long distance human movement.

### Effect of IRS on dengue infection

The odds of a secondary dengue infection at premises with confirmed dengue cases was significantly higher at unsprayed premises than at a sprayed premises (OR = 2.8; 95% CI = 1.1–6.9; *P* = 0.03). From the 151 unsprayed premises with confirmed dengue cases, 36 (23.8%) reported subsequent dengue cases at an average of 7 days (SD = 10.6 days; Max = 50 days) of the onset of symptoms of the first case in the premise. Whereas from the 97 sprayed premises with confirmed dengue cases, 13 (13.4%) reported subsequent dengue cases after the onset of symptoms of the first case in the premise. From such estimate are excluded 20 confirmed cases exposed to infective bites in sprayed premises within 7 days of an insecticide spray, and most likely infected before IRS applications. The main causes of lack of IRS applications were residents' refusal to grant access to the DART team due to personal matters or to aversion to pesticides, residents' absence at the time of DART visitation, and occurrence of locked entrance gates or dangerous dogs at a given premise. Bioassays using WHO cones on lambda-cyhalothrin-treated wood (SA Ritchie, unpublished data) together with an observed 90% reduction of gravid female collections in sticky ovitraps within a month of IRS applications in Parramatta Park [18] support the lack of resistance to lambda-cyhalothrin in local *Ae aegypti* populations.

**Figure 6:**
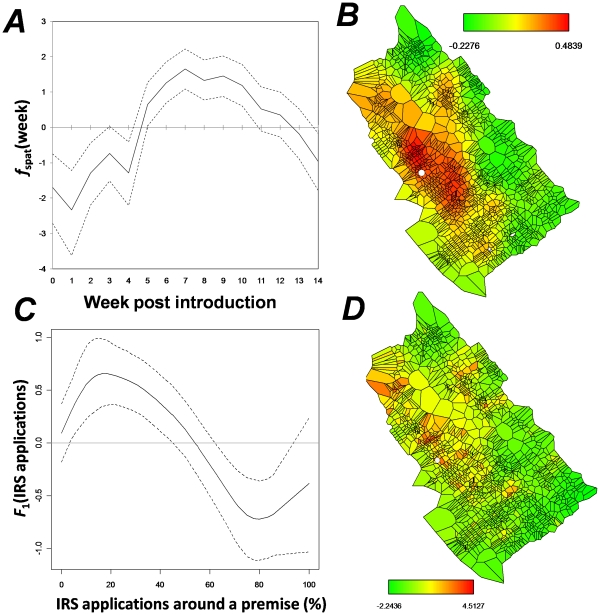
Posterior mean distributions obtained from the semi-parametric Bayesian structured additive regression (STAR) model applied to the weekly dengue infection in Cairns during the 15 weeks post virus introduction. (A) effect of time (*f*_time_) with 95% credible intervals. (B) mean posterior spatial effect (*f*_spat_). (C) effect of IRS (measured as cumulative percentage of premises sprayed around premise *i* within *t*_0_ and *t*) with 95% credible intervals. (D) mean posterior spatial effect of IRS. White dots in maps represent the location of the index case's (IC) residence.

DIC values for STAR models (2) and (3) were 2,036 and 2,025, respectively. Hence, a model without rain as a fixed effect (3) was selected as the best descriptor of the weekly spatial pattern of dengue infection. All variables in the model had an acceptance rate of 65% or more (not shown). The posterior mean (with 80% credibility intervals) of the effect of week on the odds of dengue infection is shown in Figure 6A. The odds of infection was negative from weeks 0–4 (the “silent” period before the epidemic was identified), positive from weeks 5–13 (the period of “active” transmission) and negative onwards (the period of effective control) (Figure 6A), resembling the shape of the epidemic curve (Figure 1A). The effect of the spatial occurrence of cases followed the observed space-time clustering of cases, with the highest posterior mean values (dark red in Figure 6B) found around the IC's residence and the remaining areas of medium positive (orange in Figure 6B) effect found in the periphery. The cumulative proportion of sprays around a premise had a non-linear relationship with the posterior odds of dengue infection (Figure 6C). When spraying coverage around a premise was less than 40–60%, the odds of dengue infection was positive (IRS did not prevent further cases around a premise). Only when 60% or more of the premises around a house were sprayed with insecticides the odds of infection was significantly reduced to levels below 0, and IRS applications had a protective effect in the further occurrence of dengue cases (Figure 6C). The spatial occurrence of IRS applications (Figure 6D) was similar to the distribution of cases (Figure 6B), with most of the applications in the vicinity of the IC's residence and less applications in the periphery of the introduction point. The sensitivity analysis performed by changing the hyperpriors (*a* and *b*) did not show any deviation from the basic model with default values (Figure S6 in Supporting Information S1).

## Discussion

Dengue vector control failures are partly due to our deficiencies in understanding relationships among available interventions, virus transmission dynamics, and human behavior [40]. In areas sporadically affected by epidemic dengue, such as northern Queensland, control interventions are generally implemented in response of the occurrence of local transmission, generally when it is too late to rapidly contain virus propagation [21], [22], [23], [25], [41]. By applying sound statistical analysis to a very detailed dataset from one of the largest outbreaks that affected the city of Cairns in recent times, we not only described the spread of DENV-2 with high detail but also derived important knowledge that will contribute to the understanding of how dengue virus infection propagates epidemically within complex urban environments.

Current patterns of human movement by air travel have increased the probability of introduction of dengue into *Ae. aegypti* infested areas [9]. However, it is recognized that only a handful of such introductions will have the potential of becoming IC's of a dengue epidemic. Therefore, the likelihood of a successful dengue introduction will depend on the juxtaposition of various events, such as the time a person remains viremic in the destination area (*t_v_*), the number of sites he or she visited while viremic (*l_v_*), and the number of *Ae. aegypti* bites he or she received across *l_v_* during *t_v_* [41]. Lastly, the likelihood of secondary transmission in the same area will be dependent on the exposure of local residents to infective bites from the *Ae. aegypti* females found across *l_v_* (after accounting for extrinsic incubation period and vector dispersal). All such events are significantly modulated by the impacts of vector control interventions and the build-up of population herd immunity to DENV infection occurring both in space and time. Hence, the complex pattern of dengue introduction, establishment and further propagation relies on a complex repertoire of events that, altogether, determine the spatial dimension of virus epidemic spread.

Sequential transmission (i.e., the progressive occurrence of human cases in neighboring houses), likely attributable to mosquito-driven spread and/or short-distance mobility of viremic humans, as well as long distance propagation of infection (likely generated by human mobility) have been documented in many urban dengue epidemics [21], [22], [42]. Neff et al [21] elegantly showed how, after the introduction of dengue in a Puerto Rican village, subsequent cases occurred “sequentially” within the same block and also in distant parts of the village. Similarly, phylogenetic analysis of a DENV-3 epidemic affecting the city of Sao Paulo (Brazil) allowed Mondini et al. [25] to estimate the most likely route of viral dispersal, showing that a same lineage was “dispersed” within the city both at short and long distances compatible with mosquito- and human-mediated virus spread, respectively. Our detailed analysis differentiated with great detail the contribution of short and long distance propagation of dengue virus infection within Cairns. We identified the location of 18 transmission clusters within the city that, given their space-time separation, were most likely originated by the movement of viremic humans within the city. Such clusters, although different in their extent and duration of dengue infection presence, shared a similar pattern of virus propagation from their PIdC. Outdoor sticky ovitrap collections performed in PP during the initial weeks of the epidemic evidenced high dengue virus infection rates in *Ae. aegypti* (up to 116 per 1,000), validating the occurrence of vector-mediated virus propagation within clusters. Furthermore, as most human DENV-2 infections were mild, visitation of viremic patients to their near neighbors can also help explain the rapid propagation of dengue infection within each cluster.

Build-up of immunity in the human population (*a.k.a.* herd immunity effect) has been postulated as an important driver in the dynamics of dengue virus infection, mainly by modulating human-mosquito infective contacts [5], [6]. In our study we were unable to quantitatively assess the contribution of herd effect on the local dynamics of dengue transmission (no information on human population numbers per house were available). However, given the impact and extent of the epidemic, we hypothesize that the local propagation around the IC and not the overall termination of the dengue outbreak were affected by the herd immunity effect. We base this assumption on the following observations: a) given the small and sporadic introductions of dengue, previous immunity to DENV-2 can be considered negligible, and a high population susceptibility assumed; b) a significant proportion of cases occurred in close proximity to the IC, and the ratio of infected to susceptible individuals could be high enough in such area to have a detrimental effect in the local transmission of dengue; and c) city-wide transmission was not severely impacted due to the low ratio of infected individuals to the population at risk (∼383/140,300); even assuming a 1∶10 symptomatic to asymptomatic ratio such proportion would have been lower than 5%.

The 2003 Cairns epidemic had its epicenter in the neighborhood of Parramatta Park (PP) and then progressed to the neighborhoods of Cairns North (CN) and eastern Manuda (MN). Such pattern of introduction and spread was consistent with descriptions of previous Cairns epidemics [13], [14], [17], and may be the product of the combination of demographic, environmental and entomologic characteristics unique to such areas. The high density of elevated old ‘Queenslander’ houses found in PP and CN, together with the presence of abundant tropical vegetation around them, provides optimal conditions for *Ae. aegypti* development and dispersal [18], [37]. As a consequence, such neighborhoods generally have the highest *Ae. aegypti* populations in Cairns [13], [18]. Given that most ‘Queenslander’ houses have unscreened windows, endophagy by *Ae. aegypti* can increase vector abundance but also vector-mediated virus propagation to neighboring premises (as observed in the 2003 epidemic with the propagation of infection from the IC's house –a typical ‘Queenslander’ house – to neighboring houses). Moreover, the high number of backpacker hostels found in such neighborhoods (particularly CN) increases the possibility of a dengue introduction by a viremic traveler in those areas (as observed in the 1997 Cairns epidemic that originated from a backpacker guesthouse located in CN [14], [17]). Hence, the patterns of introduction and further spread of dengue in Cairns appear to follow a consistent spatial pattern irradiating from a few key neighborhoods with particular conditions. Case detection, vector surveillance and disease management could be highly improved if such spatial heterogeneity in the likelihood of dengue introduction and spread is accounted in their design, particularly in the early stages of an outbreak.

A classic approach to reduce the propagation of infected vectors and suppress dengue transmission during the early stages of an epidemic (i.e., after the detection of active transmission) consists of the targeted implementation of vector control actions within a buffer distance (e.g., 50–100 m) of a confirmed case [43]. Such approach may be insufficient if virus human or vector propagation of dengue virus occurs beyond 100 m. Mark-release-recapture (MRR) studies have shown that urban *Ae. aegypti* females seldom disperse beyond 100 m [38], [44], [45], [46]. However, given that most mosquitoes are recaptured within 3 weeks of release, MRR studies tend to collect very limited data on the dispersal ability of older females. Thus, the contribution of older, potentially viremic, females to the tail of the dispersal distribution is highly underrepresented. By using robust space-time clustering tests we were able to use human dengue infection as a marker of virus propagation and later estimate the spatial and temporal dimensions of dengue virus spread from a putative index case (PIdC). We acknowledge that human movement may also contribute to the spread of dengue virus beyond the dispersal kernel. Nonetheless, our study showed that most (95%) of the cases associated with a PIdC were within a 200 m radius of it, and that the average spread of dengue infection varied from 14 to 32 m per week. If validated with data from other epidemics and settings, such information could have significant impacts in the design and implementation of case detection and vector control activities, particularly in areas affected by epidemic dengue. A spatio-temporal unit for case detection and control actions could help determine the area around a PIdC that would need to be screened for additional cases or controlled with insecticides or source-reduction in order to halt the propagation of dengue virus infection. Rapid (and effective) response constitutes a key operational premise in such settings [47].

Larval control and source reduction represent two of the most effective and widely used strategies to control *Ae. aegypti* populations [31], [48]. However, in areas sporadically affected by dengue epidemics preventive actions are generally rare, and most vector control activities occur upon the identification of an introduced case or the detection of active local transmission. In such circumstances, adulticiding (i.e., targeted control of adult *Ae. aegypti* by IRS or space spraying) represents an effective control action to rapidly suppress dengue transmission [48]. Additionally, where dengue hits sporadically it is common that vector control agencies are understaffed, making the response to a dengue outbreak a daunting challenge. For instance, during the 2003 epidemic Cairns Dengue Action Response Team (DART) was composed by only 4 staff members who had to be supplemented by ‘volunteers’ from other health agencies once it was evident that the number of cases exceeded the capacity of these officers to respond. Under such circumstances, our analysis shows that although IRS had a significant effect in controlling *Ae. aegypti* and reducing the odds of dengue infection, the spraying coverage around an infected premise had to be maintained at 60% or more in order to prevent subsequent infections. This finding points to the need for a coordinated effort in the design of control interventions, since partial insecticide spraying (<40% coverage) can yield to a low control effectiveness, and a higher chance of virus spread. In Cairns, lack of personnel, the occurrence of closed or conflictive premises or of property owners refusing any control activities in their houses were the main causes of the reduced insecticide coverage found in the vicinity of many dengue-positive premises. The incorporation of database and mapping technologies to track the delivery of control actions and monitor the levels of spraying coverage would prove essential to rapidly and effectively respond to a dengue epidemic [49]. Preventive control in high risk areas, capacity building (particularly in GIS/mapping and spatial analysis), incorporation of trained field technicians from other public health agencies, and integration of scientifically-based and context-sensitive control actions represent key vector control components of an integrated program geared to effectively contain dengue epidemics.

Underreporting and asymptomatic infections can represent an important proportion of the total number of dengue cases affecting a city during an epidemic [5], [6]. One of the main limitations of our study has been the lack of knowledge of the relative contribution of such “silent” infections to the total pool of cases. Dengue infection detection and notification in northern Queensland are high (due to the quality of the health system and the history of dengue introductions) [19], [24]. Hence, underreporting rates are considered low compared to the ones found in other urban centers. Nonetheless, since we analyzed data on laboratory-confirmed cases we can assume them to be the tip of the iceberg in the overall pattern of dengue spread, and that the distribution of under-reported cases will follow the one of laboratory-confirmed cases. On the other hand, the actual contribution of asymptomatic cases to the local spread of dengue is unknown, in part due to the uncertainty about their potential to infect *Ae. aegypti* mosquitoes while viremic [4]. A serologic survey or an active surveillance system could have helped assess the size of infected population (and consequently the effect of herd immunity buildup) as well as the actual attack rate and epidemic potential of dengue virus in the 2003 epidemic.

In Cairns (as in many other settings where dengue transmission is sporadic) the age-adjusted incidence was higher in young adults. Given that this age group is also one of the most mobile segments of the population their infection could have been the product of their movement (and exposure) in transmission “hot spots”. In our study we used the most likely place of transmission (instead of the case's residence) as the positional mark of a case. Such choice allowed us to more accurately analyze the propagation of dengue virus infection, since our approach included the potential exposure locations rather than merely the residential address of a case. Although TPHU nurses are highly trained and skilled in contact tracing (some of them have been performing case detection for more than 10 years), the reliability of their assessment is not perfect. However, the lack of original records of all the locations reported by a patient (due to disposal of paper records when THPU migrated to digital records) prevented us to analyze the relationship between dengue infection and the type and number of exposure locations reported and, ultimately, the accuracy of TPHU nurses in identifying transmission locations. The integration into a GIS-based decision support system of all the locations a patient has visited during the previous week to the onset of symptoms will help TPHU nurses improve their assessment. Rapid mapping of all the locations identified by the field nurses not only will allow detect where a patient may have gotten infected, but also assess which locations he or she may have visited while viremic. Such information could improve outbreak response and control of dengue epidemics.

In summary, the 2003 Cairns outbreak exhibited a pattern common to most dengue epidemic areas: virus introduction by a viremic traveler, delayed case identification and confirmation, abundant *Ae. aegypti* populations, limited (or absent) preventive control measures, delayed initiation of vector control activities once transmission was confirmed, and limited personnel/infrastructure to control a spreading epidemic. All those factors acted together to generate one of the largest outbreaks Cairns has had in recent times [13], and challenged health authorities to develop a plan to prevent epidemic spread following a point introduction. From our detailed analysis we generated a series of recommendations for TPHU that may help contain future dengue outbreaks in Cairns: a) upon suspicion of a dengue introduction, treat each potential case as dengue and perform containment activities covering all the premises found within a distance pre-spcified according to the time elapsed since the suspected introduction (as outlined in Figure 5B); b) target surveillance and preventive control in the neighborhoods dominated by older, unscreened housing such as PP and CN, that are most likely to initiate epidemic transmission; c) perform insecticide resistance tests periodically; and d) incorporate GIS and space-time analysis (by training local public health specialists) as the key tools of a decision support system for dengue management in northern Queensland. We foresee that some of the analytical procedures and derived recommendations may also be applicable to other areas currently affected or potentially subject to epidemic dengue.

## Supporting Information

Supporting Information S1 Supporting text and figures.
(2.78 MB DOC)

Video S1 Animated cartographic representation of the spread of dengue virus infection within the city of Cairns, Queensland, during the 2003 epidemic. Each frame represents a week (measured since the onset of symptoms of the introduced case, IC). Red points show the week of onset of symptoms of cases in the week t whereas blue dots the cumulative cases from t0 to t-1.
(2.89 MB WMV)
