# Supplementary material for: Quantifying the Spatial Dimension of Dengue Virus Epidemic Spread within a Tropical Urban Environment
Source: PLoS Negl Trop Dis. 2010 Dec 21;4(12):e920. doi: 10.1371/journal.pntd.0000920 (PMC3006131; doi:10.1371/journal.pntd.0000920)

**Supporting Information S1**

**Quantifying the spatial dimension of dengue virus epidemic spread within a tropical urban environment**

Gonzalo M. Vazquez-Prokopec^1,2,*^, Uriel Kitron^1,2^, Brian Montgomery ^3^, Peter Horne ^3^, Scott A. Ritchie^3,4^

*^1^Department of Environmental Studies, Emory University, Atlanta, GA, USA; ^2^Fogarty International Center, National Institutes of Health, Bethesda, MD, USA*; *^3^ Tropical Public Health Unit Network, Queensland Health, Cairns, Queensland, Australia; ^3^ School of Public Health, Tropical Medicine and Rehabilitation Sciences, James Cook University, Cairns, Queensland, Australia*

*.*

**Supplementary Text**

**The Dengue Fever Management Plan (DFMP) for North Queensland**

The operational objectives of the DFMP are : a) to recognize dengue cases as rapidly as possible through laboratory and clinical surveillance; b) to respond to dengue cases, with thorough and sustained vector control aimed at eliminating local transmission and preventing virus spread to other urban foci; c) to use a variety of education initiatives to maintain community awareness; d) to conduct preventive vector control actions in key premises like backpacker hostels and schools.

Diagnosis is performed at local laboratories using rapid immunochromatographic and enzyme-linked immunoassay (ELISA) tests to detect dengue IgM. All positive serum samples are forwarded to the reference laboratory where they are screened for the presence of anti-dengue IgM and IgG using a combined pool of flavivirus antigens in capture EIA assays. Positive IgM samples are further analyzed using flavivirus-specific IgM ELISA capture assays in order to identify the serotype of the infecting dengue virus . Additionally, real-time TaqMan reverse transcriptase-polymerase chain reaction is performed on samples collected early in the acute illness to detect dengue virus RNA .

Surveillance and control activities are dependent on the level of dengue activity . In the absence of local DENV transmission, laboratory surveillance is geared to detect imported cases. Once a locally-acquired case (i.e., an infected patient with no travel history) is confirmed, an outbreak is declared, even if only consists of a single case (the history of dengue fever outbreaks initiated by imported cases strongly supports this action ). The notification of either a dengue IgM positive test result or a suspected imported case triggers the initiation of emergency vector control activities.

Selective indoor insecticide residual spraying (SC 2.5% lambda-cyhalothrin, Demand) and larval control/source reduction activities (removal of small containers and treatment of large containers with S-methophene pellets or residual surface sprays) are performed in premises within 100 meters of a confirmed case . If multiple DENV cases are reported in a particular area, the response zone is expanded to account for virus circulation. Field data is recorded in palm-top GPS receivers (Nomad, Trimble, Sunnyvale, CA) and then imported into a Geographical Information System (GIS) for mapping vector control response activities.

**Supplementary References**

1. Health Q (2000) Dengue Fever Management Plan for North Queensland 2000-2005 [internal document]. In: TPHUN, editor. Cairns (AUST): Queensland Government. pp. 64.

2. Ritchie SA, Hanna JN, Hills SL, Piispanen JP, McBride WJ, et al. (2002) Dengue control in North Queensland, Australia: case recognition and selective indoor residual spraying. Dengue Bulletin 26: 7-13.

3. Taylor C, Simmons R, Smith I (2005) Development of immunoglobulin M capture enzyme-linked immunosorbent assay to differentiate human flavivirus infections occurring in Australia. Clin Diagn Lab Immunol 12: 371-374.

4. Warrilow D, Northill JA, Pyke A, Smith GA (2002) Single rapid TaqMan fluorogenic probe based PCR assay that detects all four dengue serotypes. J Med Virol 66: 524-528.

5. Hanna JN, Ritchie SA (2009) Outbreaks of dengue in north Queensland, 1990-2008. Commun Dis Intell 33: 32-33.

**Supplementary Figures**

**Figure S1.** Form used by Queensland’s medical general practitioners reporting suspected or confirmed cases to TPHU. Forms are submitted by fax and processed by TPHU immediately after receipt.

**
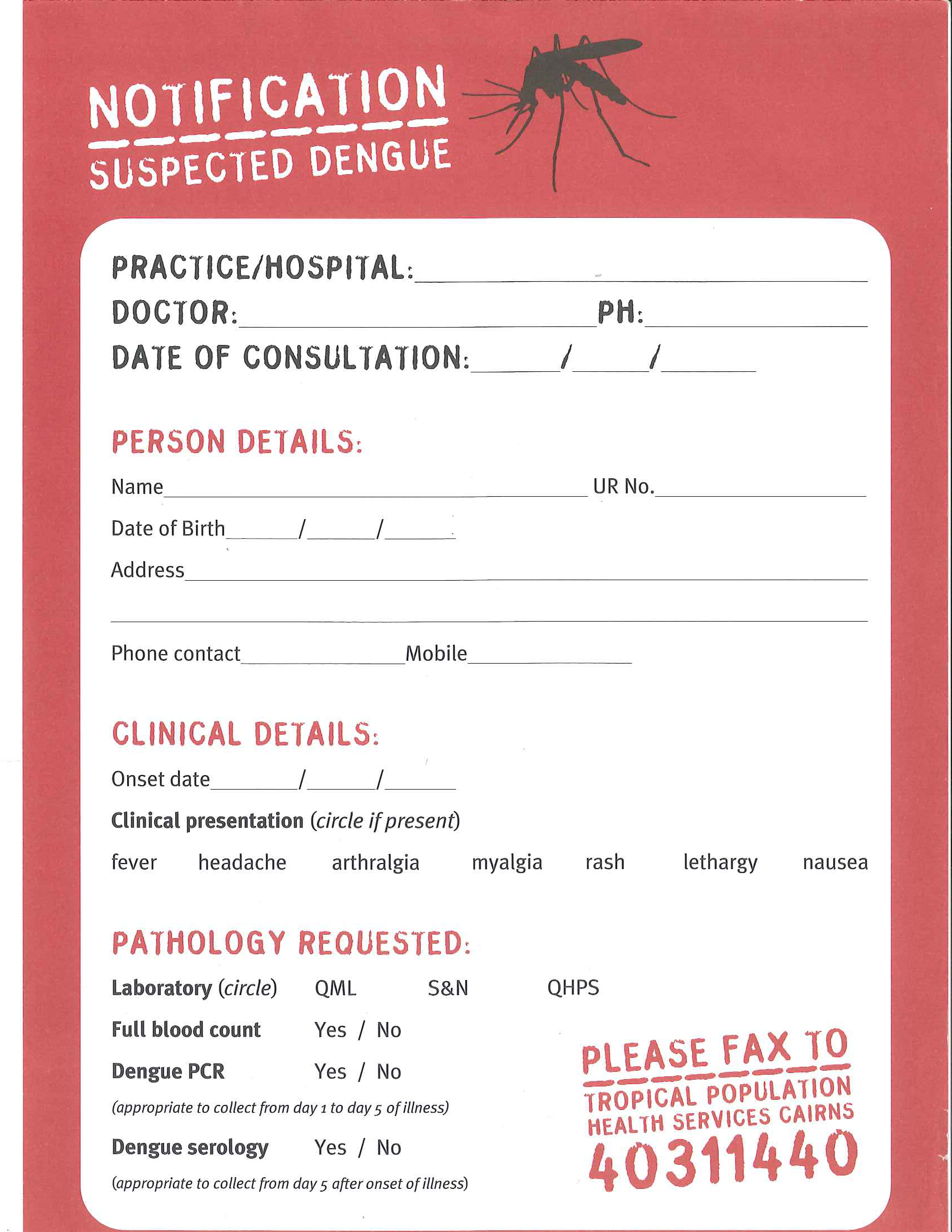
**

**Figure S2.** Dengue case report forms used by TPHU public health nurses to interview suspected or confirmed dengue cases (and their contacts) and ascertain the locations visited while viremic and, ultimately, the most likely place of transmission (called acquired where in the form).

**
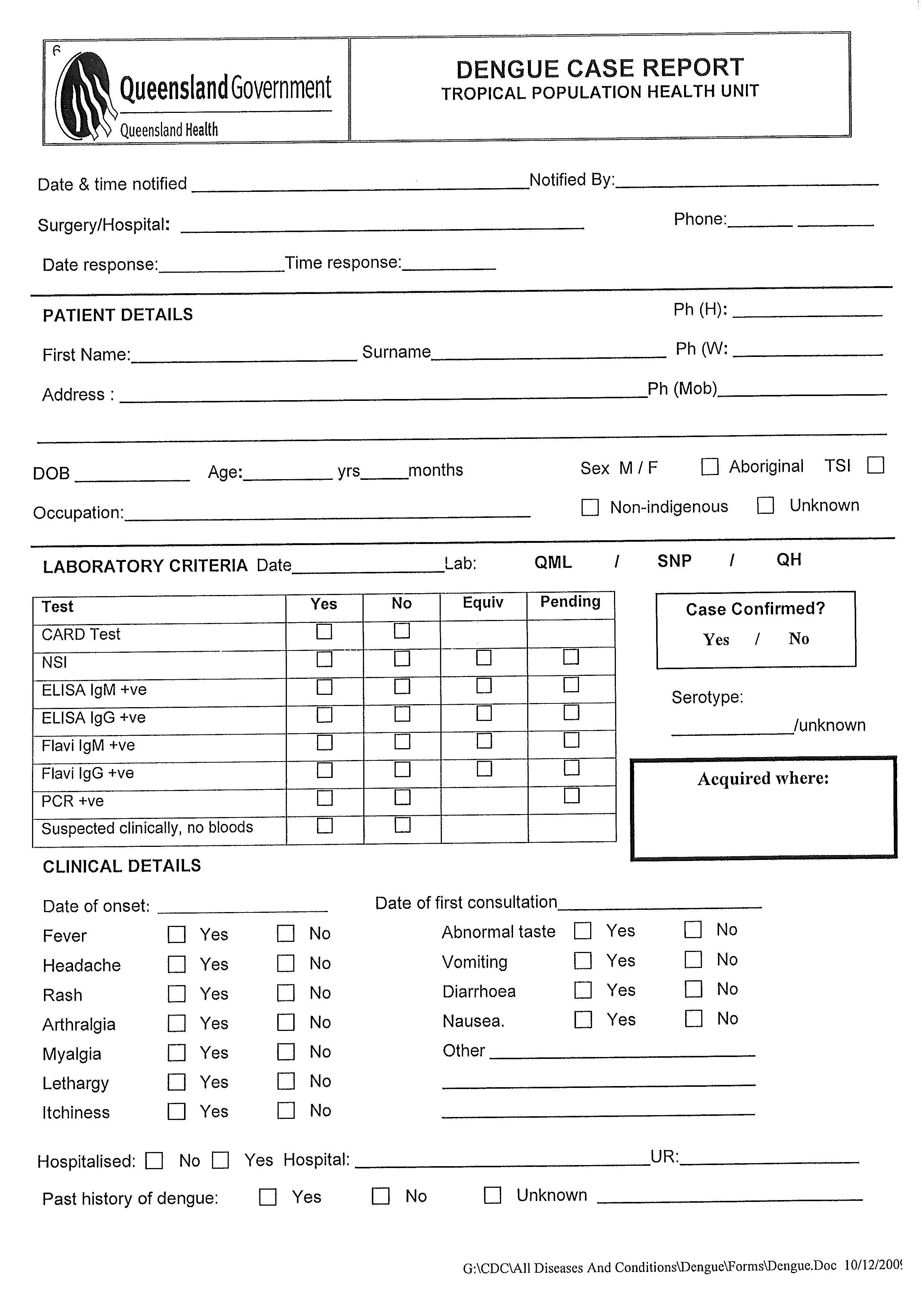

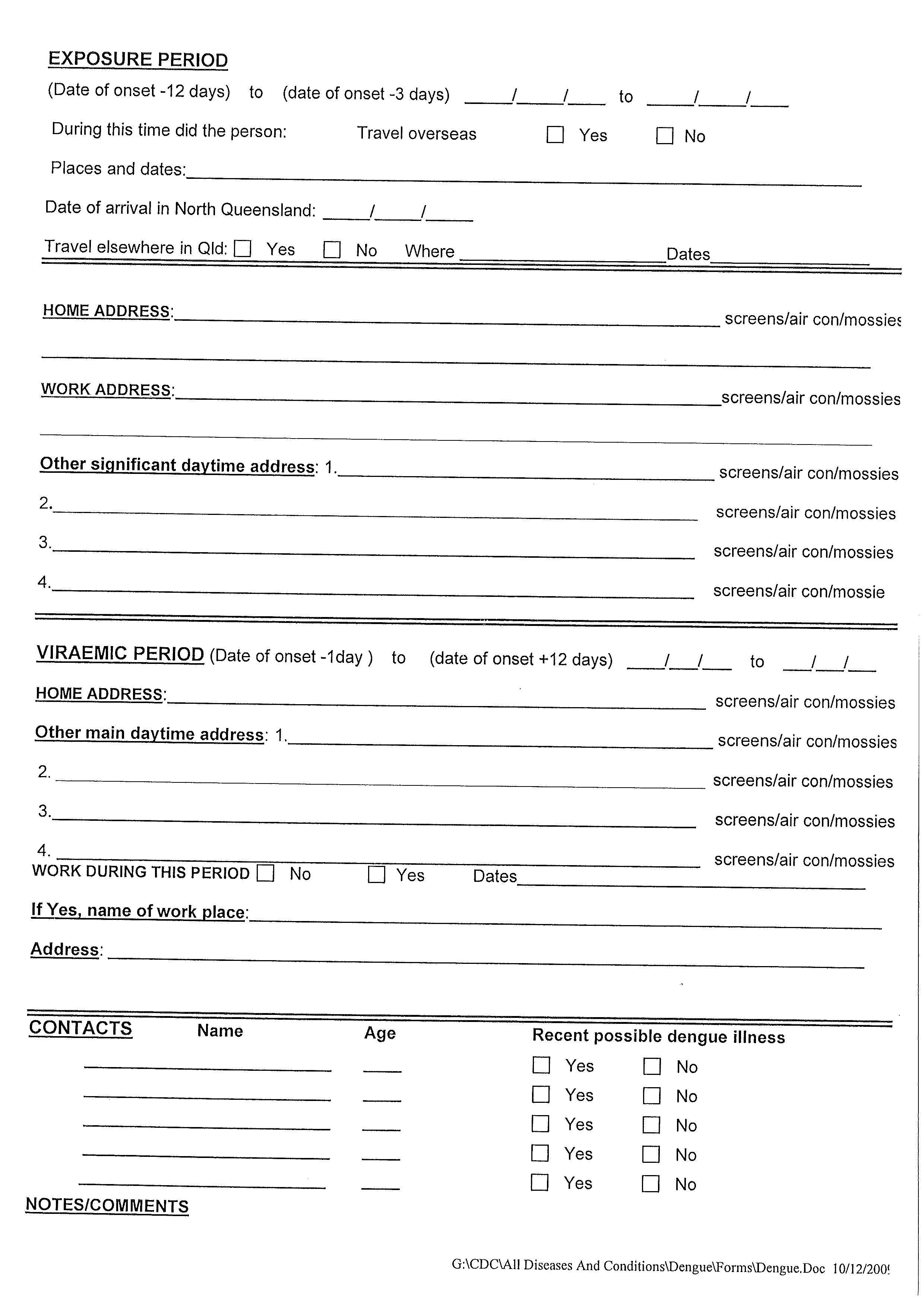
**

**Figure S3.** Cross-correlations between the number of dengue cases occurring at a given week and the weekly number of IRS applications, total rain, mean temperature, and mean relative humidity registered in Cairns during January-August 2003. The larger the bar the higher the correlation between variables observed at that time lag.

**Figure S4.** Focal clustering of dengue cases around the introduced case (IC). (A) Spatial correlogram showing the distance up to which clustering occurred (*d_max_*). (B) Map showing the extent of *d_max_*.


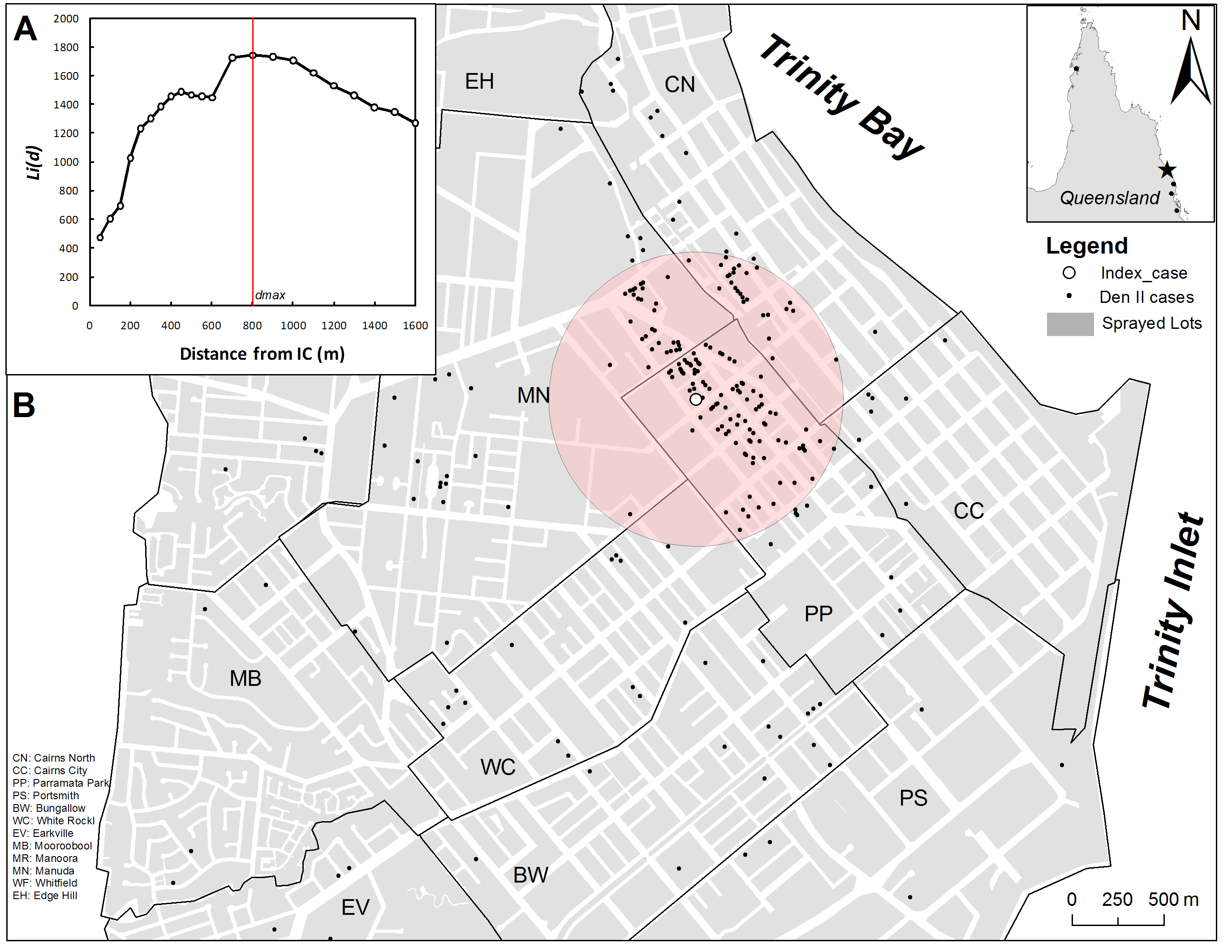


**Figure S5.** Proportion of total cases per week on each space-time cluster identified in the city of Cairns during the 2003 dengue epidemic.


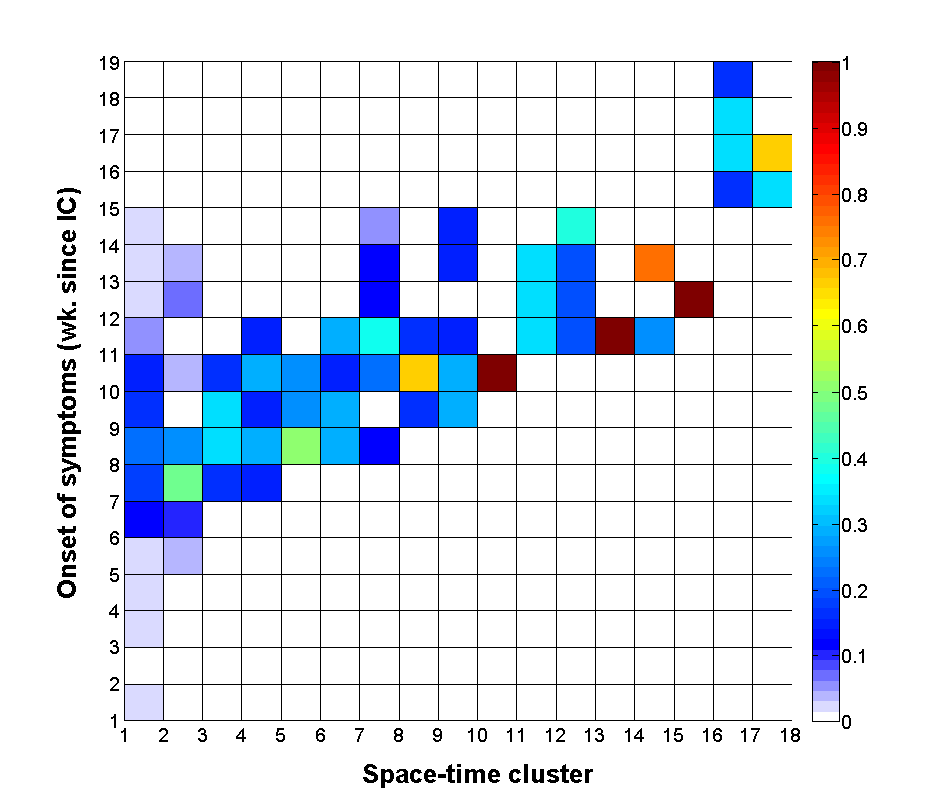


**Figure S6.** Sensitivity analysis of the Bayesian structured additive regression model (STAR) applied to assess the relationship between IRS and the odds of dengue infection. Plotted are the posterior means of (A) effect of time (*f*_time_) with 80% credible regions. (B) mean posterior spatial effect (*f*_spat_). (C) effect of IRS (measured as cumulative % of premises sprayed around premise *i* within *t*_0_ and *t*). (D) mean posterior spatial effect of IRS for high (left) and low (right) hyperparameter (*a* and *b*) values.


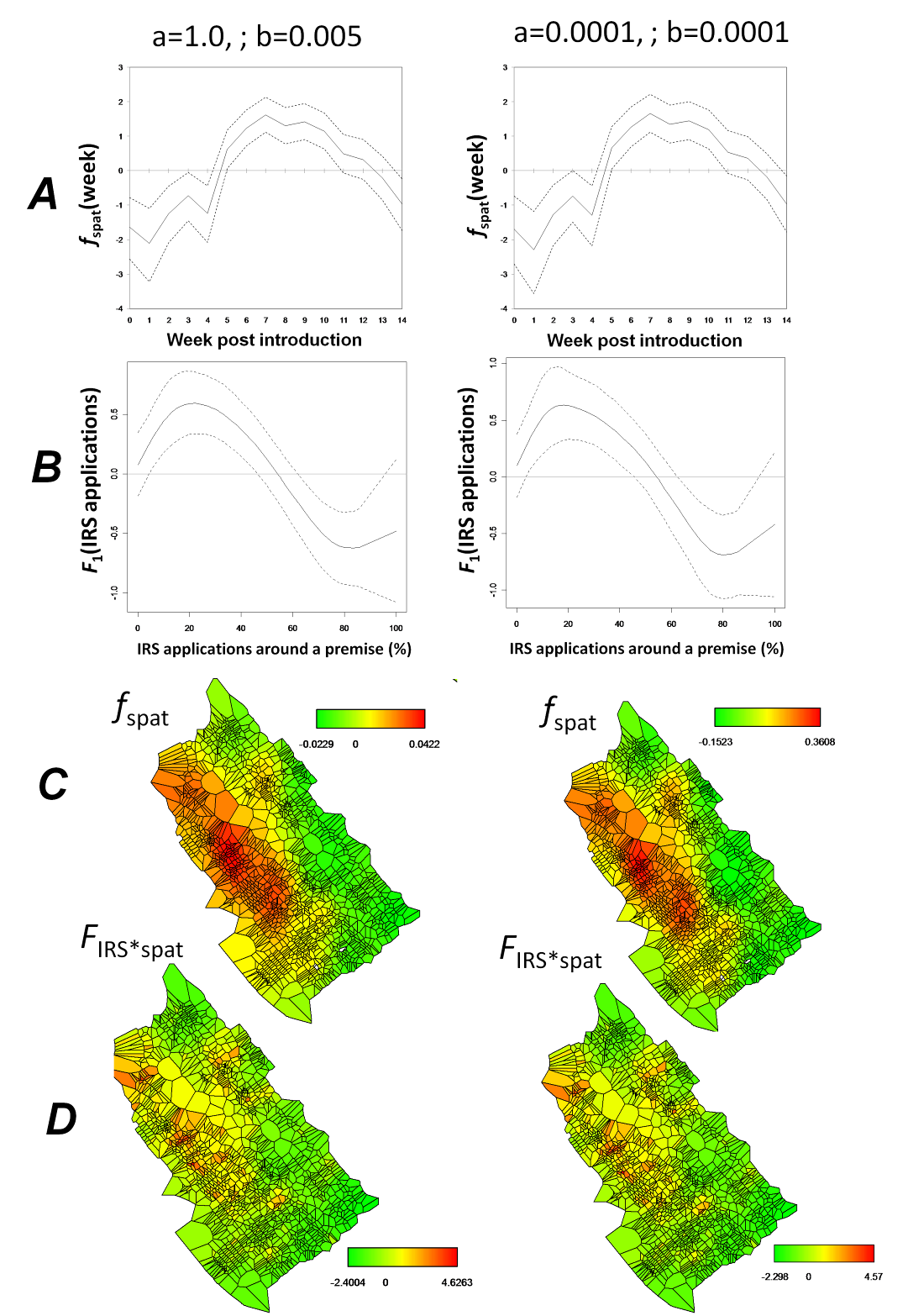

Supplement: Supporting Information S1 — Supporting text and figures. (2.78 MB DOC) [file pntd.0000920.s001.doc]
